# Application of deep learning for the analysis of stomata: a review of current methods and future directions

**DOI:** 10.1093/jxb/erae207

**Published:** 2024-05-08

**Authors:** Jonathon A Gibbs, Alexandra J Burgess

**Affiliations:** Agriculture and Environmental Sciences, School of Biosciences, University of Nottingham Sutton Bonington Campus, Loughborough LE12 5RD, UK; Agriculture and Environmental Sciences, School of Biosciences, University of Nottingham Sutton Bonington Campus, Loughborough LE12 5RD, UK; University of Essex, UK

**Keywords:** Deep learning, gas exchange, object detection, photosynthesis, semantic segmentation, stomata, water use

## Abstract

Plant physiology and metabolism rely on the function of stomata, structures on the surface of above-ground organs that facilitate the exchange of gases with the atmosphere. The morphology of the guard cells and corresponding pore that make up the stomata, as well as the density (number per unit area), are critical in determining overall gas exchange capacity. These characteristics can be quantified visually from images captured using microscopy, traditionally relying on time-consuming manual analysis. However, deep learning (DL) models provide a promising route to increase the throughput and accuracy of plant phenotyping tasks, including stomatal analysis. Here we review the published literature on the application of DL for stomatal analysis. We discuss the variation in pipelines used, from data acquisition, pre-processing, DL architecture, and output evaluation to post-processing. We introduce the most common network structures, the plant species that have been studied, and the measurements that have been performed. Through this review, we hope to promote the use of DL methods for plant phenotyping tasks and highlight future requirements to optimize uptake, predominantly focusing on the sharing of datasets and generalization of models as well as the caveats associated with utilizing image data to infer physiological function.

## Teaser

This review discusses the application of deep learning approaches for the assessment of stomata, including variations in the pipeline used, from data collection to parameter extraction.

## Introduction

An increasing population and corresponding increasing demand for food is putting pressure on farmers and breeders to ensure future food security goals are met. This is exacerbated by climate change projections, which indicate increased warming and drying trends for the upcoming decades (IPCC, 2022). Crop yield largely depends on the cumulative rate of photosynthesis as well as the availability of water. Therefore, optimizing both photosynthesis and water use efficiency, the balance between carbon gain and water lost, is a key target for crop improvement (Long *et al.*, 2006; Furbank *et al.*, 2015; Condon, 2020).

**Fig. 1.**
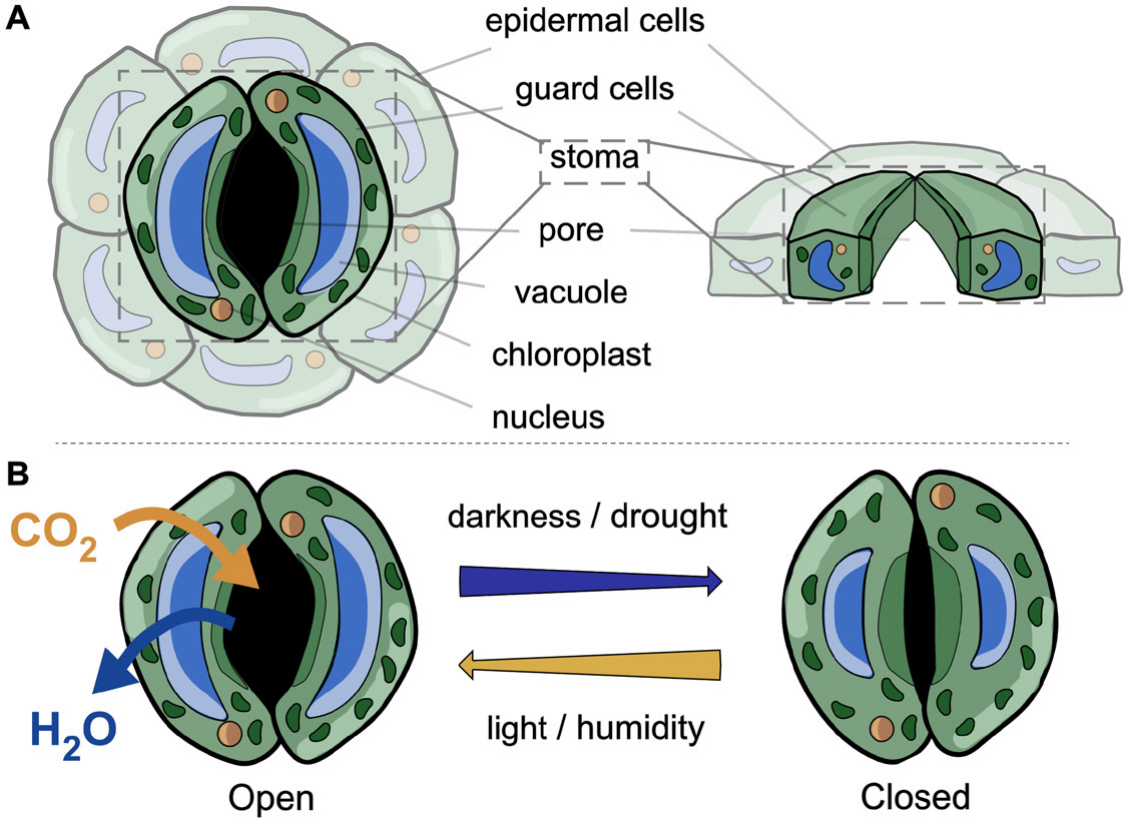
Diagram of stomatal structure and function in facilitating gas exchange—example of a dicot stoma. (A) Surface and transverse view of a stoma, encompassing the guard cells and pore, as denoted by the box, and accompanying epidermal cells (faded out). (B) Internal and external signals confer a structural change in the stoma to permit gas exchange when the structure is open, and restrict exchange when closed.

As gatekeepers between the plant and its environment, stomata (singular ‘stoma’) play a pivotal role in determining physiological function and metabolism. Here, we refer to stomata as the combination of guard cells and the pore, regardless of whether they are ‘open’, where the swelling of guard cells increases the size of the pore, or ‘closed’, where guard cells shrink and pore area reduces (Fig. 1). Although stomata occupy only 0.3–5% of the leaf epidermal surface, they account for up to 98% of gas exchange (Lawson and Blatt, 2014). The appearance of stomata varies across species, with guard cells that are dumbbell shaped in monocot grasses, to kidney shaped in the dicots. Guard cell morphometry and stomatal density (the number of stomata per unit area) are anatomical features that are usually defined during organ development and provide routes to altering plant metabolism (e.g. Franks *et al.*, 2015).

Stomatal traits can be measured using direct or indirect approaches (Beadle *et al.*, 1985). The former generally encompasses image-based approaches, and enable the analysis of shape, size, and orientation of stomata. These morphometric measures are important to support the analysis of photosynthesis, which is limited by those traits. In comparison, indirect approaches, such as the use of porometers, infrared gas analysers, or leaf temperature measurements, informs the function of stomata including conductance capacity and opening and closure dynamics (e.g. Ceciliato *et al.*, 2019). For a full understanding of plant–environment interactions, a combination of both morphometry and functional assessment is required.

The analysis of stomata is a long-standing research area (Joseph, 1805); nonetheless, as recently as 2017 biologists had few tools to automatically analyse images containing stomata, instead relying on manual, labour intensive, and error prone methods to extract features. With increases in the accessibility and affordability of computing power, recent years have seen a boom in the application of deep learning (DL) models (see Box 1) for plant physiological analysis, including the assessment of stomata (Thompson *et al.*, 2017; Balacey *et al.*, 2023, Preprint). Various DL models have been proposed, permitting the rapid detection of stomata and thus providing a platform for automated high-throughput analysis. Most commonly, particularly in the stomata literature, DL methods can be broadly categorized as: (i) object detection, which estimates localization and class of an object within a given image, encapsulating it within a box, and (ii) semantic segmentation, which operates at pixel level classifying each individual pixel, for example whole stomatal complex, pore, guard cell, or background (Zhao *et al.*, 2019; Minaee *et al.*, 2022). Semantic methods provide finer-grained information with respect to object detection by detecting object boundaries, therefore preserving morphology. However, these methods tend to be more computationally expensive, require larger annotated datasets, and are more sensitive to changes in environmental conditions. Additionally, though rarely seen in stomata literature, there is (iii) instance segmentation, which identifies the different instances of the same class at pixel level (Hafiz and Bhat, 2020). Ultimately, choosing between each of the model types depends on the required level of detail; for example, counts and density would be more suitable for object detection, as opposed to finer details such as lengths and areas, which require semantic segmentation.

With this paper, we review current publications that apply DL to the analysis of stomata. We discuss the different pipelines to obtain image data, common preprocessing steps, differences in the main network structures and the outputs, and post-processing steps that lead to stomatal trait measurement. We hope to provide an insight into available methods and applications as well as the future direction for DL-based analysis of stomatal traits. Through this review, we hope to encourage the uptake of deep learning for analysis of stomata and facilitate the first step towards improved collaborative working and publication of a global dataset.

Box 1. **Overview of deep learning and convolutional neural networks**

Deep learning (DL) is a form of machine learning that teaches computers to process data similarly to the human brain (Pound *et al.*, 2016). DL models are trained to recognize complex patterns and to produce accurate insights and predictions, automating tasks that typically require human intelligence.

A convolutional neural network (CNN) is a type of DL network that is optimized to work with image, or pixel level, data (Rawat and Wang, 2017). A CNN takes an image as an input, passes it through the contained layers, and outputs a prediction that represents the class data designated in the training set. As such, CNNs act as basic building blocks for the computer vision task of image recognition and segmentation. They consist of a varying number of layers, each of which has trainable parameters. Common layers include:

(i) Convolutional layers, which use filters and kernels to produce a more abstract representation via a feature map. These filters aim to detect patterns such as edges. The filter passes over the image like a scanner and creates a feature map.
(ii) Pooling layers act down sample feature maps by summarizing the presence of features in patches of the feature map. This reduces the dimensionality of the data, with a corresponding reduction in computational cost.
(iii) Fully connected layers connect neurons in one layer to neurons in another layer. This takes the outputs from other layers and classifies pixels, computing scores for each of the class labels.

The structure of a CNN will vary depending on the data used, the application, and the size of the network. This leads to a variety of possible network structures.

## Pipelines of stomatal analysis

**Fig. 2.**
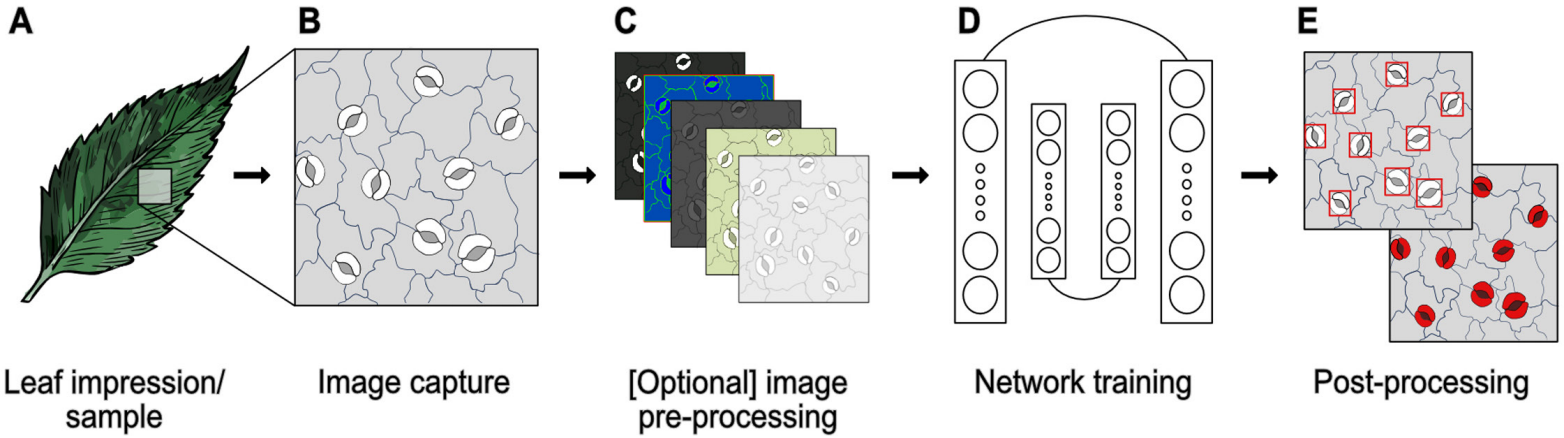
Overview of the pipeline for the assessment of stomata. (A) data acquisition encompassing either leaf sampling or taking surface impressions, (B) image capture, (C) optional pre-processing of image data, (D) training of a deep learning model, or application of a pre-trained model, and (E) post-processing of network outputs.

Extracting stomatal morphometry using DL can be broadly classified into four processes: data acquisition, pre-processing, deep learning and evaluation, and post-processing (Fig. 2). Variation exists for each of these steps, with the most common methodologies discussed in more detail in the following sections.

### Data acquisition

Image acquisition of stomata (Fig. 2A, B) can be classified into two broad approaches: destructive and non-destructive methods. The former damages the leaf material impacting functionality or future measurements whilst the latter preserves the leaf in its current state. The choice of data acquisition depends upon numerous considerations including plant species, hardware access, and study aim. Certain plant characteristics, such as thick wax layers, cuticle, or trichomes that protect the epidermal layer, may limit the visibility of stomata in some cases.

The most common method to capture image data is using leaf impressions. Silicone, dental resin, and/or nail varnish can be used in isolation or combination to capture surface structure (Gitz and Baker, 2009). Whilst these methods are most commonly cited in the literature, including for the training of DL models (see below), it is widely accepted that leaf impressions provide an accurate estimate of stomatal density, but permit considerable error when estimating pore or stomatal complex dimensions (Matthaeus *et al.*, 2020). An alternative non-destructive approach is the use of handheld microscopes to directly image the plant surface *in situ* (Pathoumthong *et al.*, 2023). If captured via video format, this permit the additional analysis of stomatal behaviour, such as dynamic changes in aperture size.

Destructive methods can be used to maximize visibility of stomatal structure, and can help to overcome problems associated with artefacts in image data. This often relies on methods to clear the tissue of pigments and/or enhance certain structures using stains.

### Pre-processing image processing and data annotation

Pre-processing of image data constitutes an optional step to improve image quality or data consistency prior to analysis. Pre-processing may include image processing methods such as contrast-limited adaptive histogram equalization (CLAHE), noise reduction, and manual editing of data (Fig. 2C).

For DL application, a series of manual measurements or annotations must be made to obtain a ground truth for training. Annotations can be made using freely available software such as LabelImg (https://github.com/tzutalin/labelImg), which is popular for annotating bounding boxes, and PixelAnnotationTool (Bréhéret, 2017) for semantic segmentation. Whilst larger datasets are often the most desirable option, this is not always feasible and instead methods to increase the size of small datasets are often used. A common approach is to use augmentations, applying operations such as blur, flip, and rotate to images (Gibbs *et al.*, 2019, 2021). This usually occurs after annotation to save time. Such approaches can aid in alleviating overfitting, where the DL model tries to entirely fit the training data and so cannot be readily applied to new, unseen data. Additionally, generative adversarial networks (GANs) can be used to generate artificial data, though this requires an initial set of images to train.

### Deep learning

Whilst an in-depth insight into each of the deep learning architectures is out of the scope of this paper, we do provide an overview of common networks and corresponding publications relevant to stomata in Table 1. All of these models take the form of convolution neural networks (CNNs; Box 1). These cover both object detection (e.g. AlexNet, YOLO, SSD, R3DET, VGG, R-CNN, and MobileNet) and semantic segmentation methods (e.g. Mask R-CNN and UNet).

**Table 1.** An overview of the main deep learning networks applied to stomata analysis

Name	Type	Description	Papers Inc.
AlexNet	Object detection	AlexNet, a CNN with eight layers, is primarily used for classification and recognition. It is considered one of the most influential papers published in computer vision and was heavily behind the surge in DL approaches for vision tasks being the first to employ a CNN on a GPU	Millstead *et al.* (2020)
YOLO	Object detection	YOLO, often used for real time detection, is one of the most popular DL models due to its speed and accuracy. YOLO predicts localization and class probabilities simultaneously. Several versions of YOLO exist including those that can be used in combination with segmentation algorithms	Casado-García *et al.* (2020), Ren *et al.* (2021), Sultana *et al.* (2021), Yang *et al.* (2021), Dai *et al.* (2022, Preprint), Zhang *et al.* (2022), Li *et al.* (2023), Pathoumthong *et al.* (2023), Takagi *et al.* (2023), Wang *et al.* (2024a)
SSD	Object detection	SSD is much like YOLO in that it only takes a single pass for detecting objects within in image and does not use region proposal, one of the primary reasons for its speed and efficiency	Toda *et al.* (2021)
R3Det	Object detection	R3Det is a refined single-stage detector rotation detector for fast and accurate object detection by using a progressive regression approach. It works much like YOLO and SSD in that it only uses a single stage; however it aims to address the issues relating to misalignment of objects	Yang *et al*. (2024)
VGG	Object detection	VGG is a standard deep CNN which specializes in localization and classification of objects. Two popular VGG architectures exist, VGG-16 and -19, where the numbers correspond to the number of layers within the architecture.	Sakoda *et al.* (2019), Meeus *et al.* (2020), Aono *et al.* (2021)
R-CNN	Object detection	R-CNN is used for classifying and localizing objects. It is a two-stage object detection model, proposing a series of regions and then evaluating these, determining which class the region lies in. R-CNN has multiple variations though the most common are Basic R-CNN, Fast R-CNN, and Faster R-CNN	Li *et al.* (2019), Costa *et al.* (2021), Cowling *et al.* (2021, Preprint), Zhu *et al.* (2021), Liang *et al.* (2022)
Mask R-CNN	Semantic	Mask R-CNN extends Faster R-CNN by adding an additional operation at the end to predict the object mask. It is a semantic and instance segmentation technique that performs pixel-level segmentation on detected objects	Song *et al.* (2020), Bheemanahalli *et al.* (2021), Costa *et al.* (2021), Jayakody *et al.* (2021), Xie *et al.* (2021), Sai *et al.* (2022, Preprint), Meng *et al.* (2023)
MobileNet	Object detection	MobileNet is based on a streamlined architecture that uses depth-wise separable convolutions to build lightweight networks designed for mobile and embedded vision applications. Particularly beneficial when computing power is lacking or unavailable	Kwong *et al.* (2021), Razzaq *et al.* (2021)
U-Net	Semantic	U-Net, originally introduced for medical imaging, typically requires less training data than other methods to achieve similar results. It produces pixel-wise segmentation and classification	Gibbs *et al.* (2021), Sun *et al.* (2021, 2023), Takagi *et al.* (2023), X. Zhang *et al.* (2023, Preprint)
Custom CNN	Multiple	Custom CNN refers to individually made CNNs that combine a series of convolutions, pooling, and fully connected layers. Each differ quite significantly, so refer to each individual paper for a more in-depth overview. Custom CNNs can have any desired output but often require extensive expertise. In the papers cited here, outputs were in the form of image classification, heatmaps, and 2D points	Jayakody *et al.* (2017), Bhugra *et al.* (2018, 2019), Toda *et al.* (2018, Preprint), Fetter *et al.* (2019), Andayani *et al.* (2020), Hunt *et al.* (2021), Li *et al.* (2022), Dey *et al.* (2023), F. Zhang *et al.* (2023)

CNN, convolutional neural network; DL, deep learning; R-CNN, region-based convolutional neural network; SSD, single shot detector; VGG, visual geometry group; YOLO, you only look once.

For all DL models, annotated data are split into train and test data, commonly at a 4:1 ratio. The training data are used to train the selected network (Fig. 2D; Table 1), whilst the test data are used to evaluate the performance of the network. The amount of data required will depend on the network selected, the variability of the data set, and the number of features present per image.

The performance of DL can be evaluated by a variety of methods, the most common of which are discussed in Box 2. For semantic segmentation, common evaluation metrics include pixel accuracy and mean pixel accuracy. F1 score, precision, recall, accuracy, and intersection over union apply to both semantic and object detection- based architectures, whilst mean average precision applies only to object detection-based architectures. Whilst evaluation metrics provide a good indication of performance on the dataset in question, the same evaluation metrics from different networks are not comparable to each other unless the same dataset has been used. Similarly, the biological insight that can be obtained from a DL model relies on the accuracy or validity of the original data. For example, combining datasets collected using different data acquisition methods requires consideration of the potential errors associated with each method.

Box 2. **Evaluation methods for deep learning architectures**

Deep learning (DL) models can be evaluated using different metrics that enable a quantitative measure of the performance and effectiveness the given model. For semantic segmentation, metrics such as pixel accuracy (PA) and mean pixel accuracy (mPA) can provide insight into the accuracy of pixel predictions. PA denotes the percentage of correctly predicted pixels:

$$\begin{array}{l} PA=\frac{\overset{k}{\underset{i=0}{\sum }}p_{ii}}{\overset{k}{\underset{i=0}{\sum }}t_{i}}\;\;\; \end{array}$$ (1)

where $p_{ii}$ is the total number of pixels both classified and labelled as class i and $t_{i}$ is the total number of pixels labelled as class i.

Semantic segmentation deals with a minimum of two classes and therefore mPA is often used to represent the class accuracy:

$$\begin{array}{l} mPA=\frac{1}{k}\underset{i=0}{\overset{k}{\sum }}\;\frac{p_{ii}}{t_{i}}. \end{array}$$ (2)

However, it is worth noting that a high-class accuracy does not always guarantee superior performance if it is at the expense of other classes.

F1-score, precision and recall are evaluation metrics, used for both semantic and bounding box models. Evaluation is based on true positives (TP), where the model correctly predicts the positive class; true negatives (TN), where the model correctly predicts the negative class; false positives (FP), where the model incorrectly predicts the positive class; and false negatives (FN), where the model incorrectly predicts the negative class. Precision is the ratio of correct annotations relative to the total number of annotations (true and false positives):

$$\begin{array}{l} precision=\frac{TP}{TP+FP}. \end{array}$$ (3)

Recall is the ratio of correct annotations relative to the total number of ground truth annotations (true positives and false negatives):

$$\begin{array}{l} precision=\frac{TP}{TP+FP}. \end{array}$$ (4)

F1-score is the harmonic mean of precision and recall, allowing a balance between the two, thus providing a greater insight into the measure of incorrect annotations:

$$\begin{array}{l} F1=\frac{2\times \left(precision\times recall\right)}{precision+recall} \end{array}$$ (5)

Accuracy describes how the model performs across all classes, calculated as the ratio between the number of correct predictions to the total number of predictions:

$$\begin{array}{l} Acc=\frac{TP+TN}{TP+TN+FP+FN} \end{array}$$ (6)

The intersection over union (IoU) is a number between 0 and 1 that specifies the amount of overlap between predicted and ground truth (i.e. manual) annotations. A value of 0 indicates there is no overlap, whilst 1 indicates a perfect union of ground truth and prediction.

$$\begin{array}{l} IoU=\frac{Area\;of\;overlap}{Area\;of\;union} \end{array}$$ (7)

For object detection methods only, mean average precision (mAP) is a common evaluation metric calculated using IoU, a confusion matrix (including TP, FP, FN), precision, and recall:

$$\begin{array}{l} mAP=\frac{1}{n}\;\underset{n=1}{\overset{k=n}{\sum }}\;AP_{k} \end{array}$$ (8)

Where AP_k_ is the average precision of class *k* and *n* is the number of classes.

### Post-processing

**Fig. 3.**
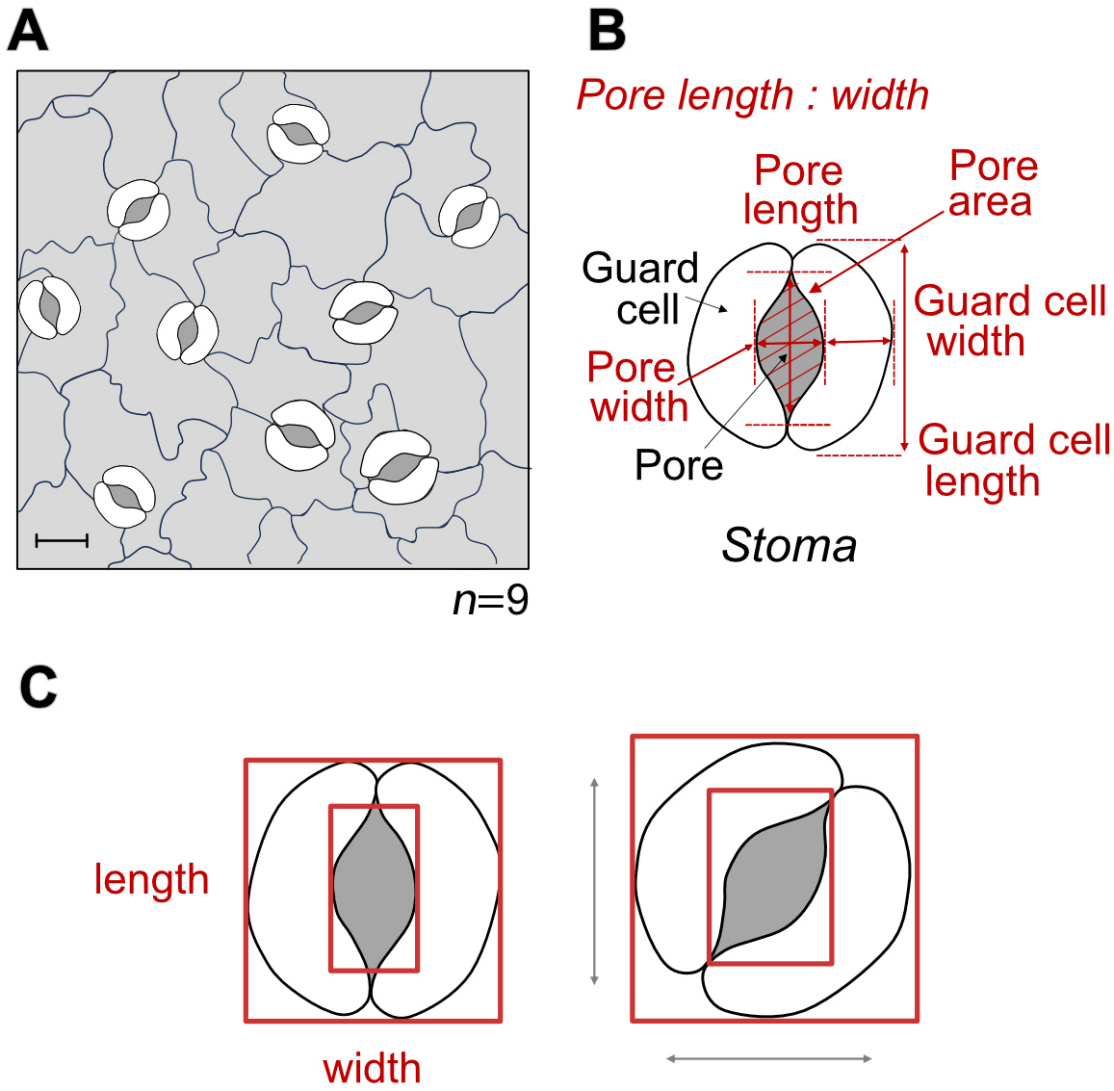
Overview of typical measurements performed on image data containing stomata—example of a representative dicot leaf. (A) Detection of stomata in images can be used for counting stomata or assessment of stomatal density. (B) Extraction of individual stoma can be used to calculate morphometry measurements including areas and dimensions. (C) Depending on the deep learning network used, bounding box methods may lead to incorrect morphometry measurements if the stomata are not orientated along the major axes. Note that this diagram has been slightly re-sized to emphasize the difference.

Post-processing is performed on the output of the trained DL model. High throughput methods aim to automate the estimation of stomatal morphometry (Fig. 3) through various post-processing steps; these include operations such as ellipse fitting, level set methods, and contour extraction to attempt to fine tune the stomata, guard cell, or pore perimeter. Alternatively, methods such as blob detection can be used for counting and estimating density. Additionally, calculations may be performed, for example estimating conductance (e.g. Gibbs *et al.*, 2021), with the results output into a readable format.

## Published deep learning methods for the study of stomata

**Fig. 4.**
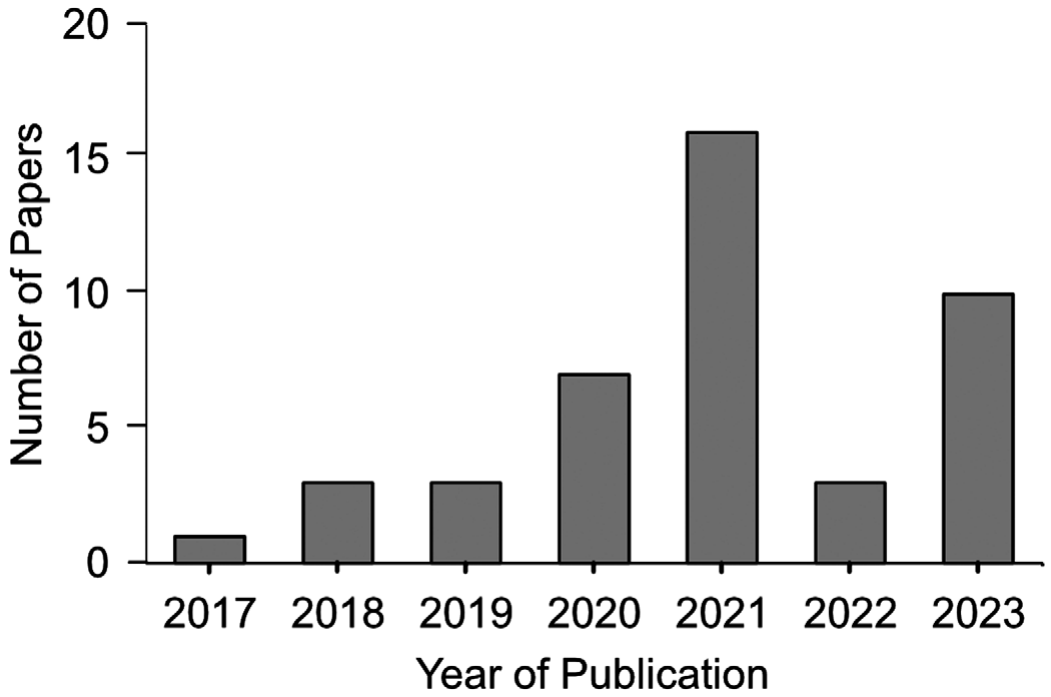
Bar graph presenting the number of deep learning publications applied to stomata over the last 7 years.

**Fig. 5.**
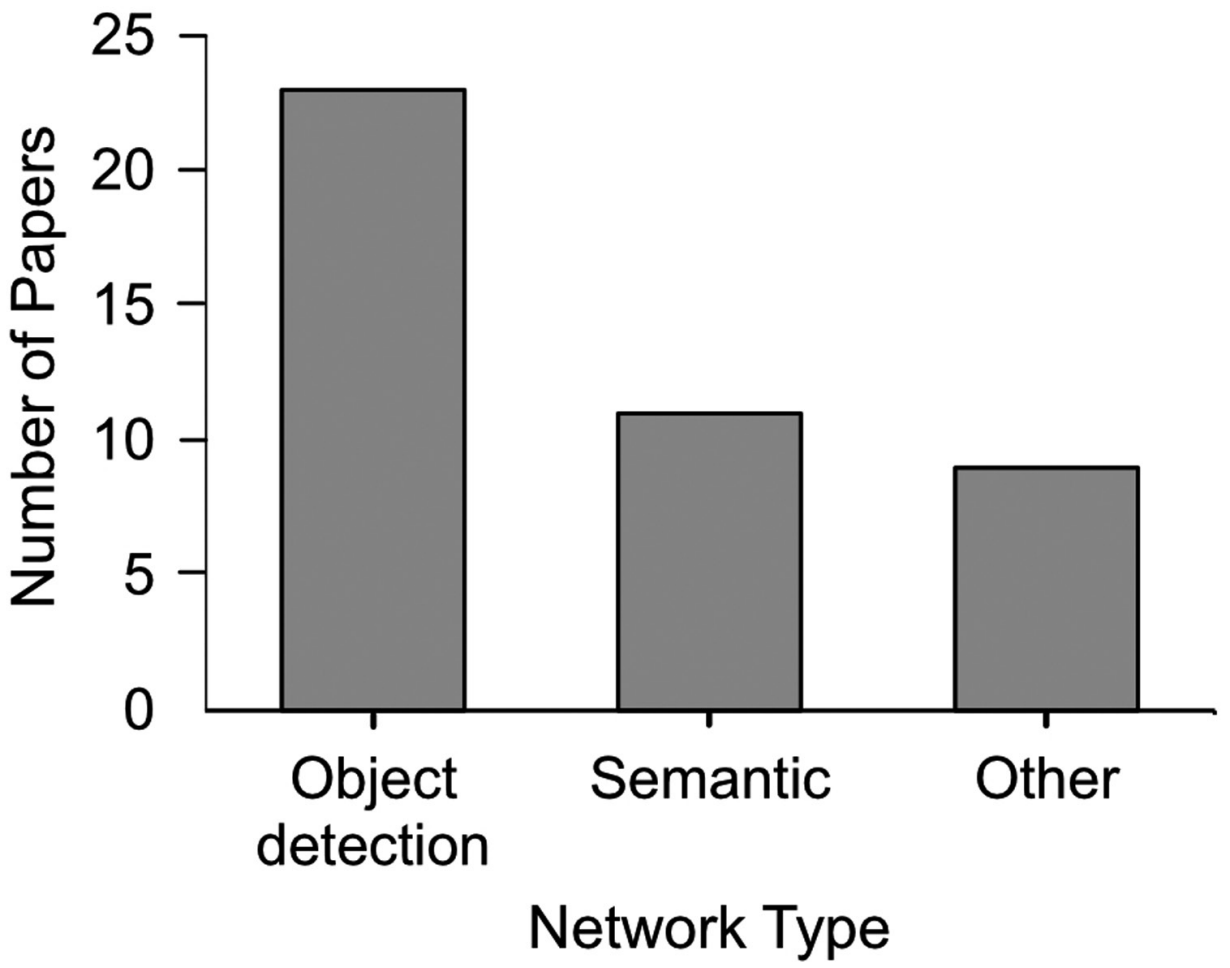
Bar graph presenting the breakdown of deep learning network types applied to stomata.

A review of the literature indicated a total of 43 publications that employed deep learning methods to the assessment of stomata over a 6-year period (2017–2023), covering ~25 species, or phylogenetic groups, of plants (Table 2). The number of publications has steadily risen per year, with a peak in papers during 2021 (Fig. 4). These are diverse, encompassing DL approaches for object detection based on bounding boxes, sematic segmentation, and/or other custom outputs (Fig. 5). Furthermore, the methods used to capture the initial datasets are diverse, although the majority of papers use nail varnish-based surface impressions (Table 3).

**Table 2.** Overview of plant species that have been studied using deep learning approaches to analyse stomatal traits

Type	Paper
Apricot	Millstead *et al.* (2020)
Arabidopsis	Li *et al.* (2022), Sai *et al.* (2022, Preprint), Takagi *et al.* (2023), Yang *et al*. (2024)
Barley	Casado-García *et al.* (2020), Hunt *et al.* (2021), Sai *et al.* (2022, Preprint)
Broadbean	Li *et al.* (2023)
Common bean	Casado-García *et al.* (2020)
Dayflower	Toda *et al.* (2018, Preprint)
Gingko	Fetter *et al.* (2019), Jayakody *et al.* (2021)
Grapevine	Jayakody *et al.* (2017), Millstead *et al.* (2020)
Hardwood trees	Wang *et al.* (2024b)
Haskap	Meng *et al.* (2023)
Herbarium samples	Meeus *et al.* (2020)
Lettuce	X. Zhang *et al.* (2023, Preprint)
Maize	Aono *et al.* (2021), Ren *et al.* (2021), Xie *et al.* (2021), Yang *et al.* (2021, 2024), Liang *et al.* (2022), F. Zhang *et al.* (2022, 2023)
Oil palm	Kwong *et al.* (2021)
Orange	Millstead *et al.* (2020), Costa *et al.* (2021)
Periwinkle	Millstead *et al.* (2020)
Poplar	Li *et al.* (2019), Song *et al.* (2020), Gibbs *et al.* (2021), Jayakody *et al.* (2021), Dai *et al.* (2022, Preprint), Wang *et al.* (2024a)
Quinoa	Razzaq *et al.* (2021)
Rice	Bhugra *et al.* (2018, 2019), Cowling *et al.* (2021, Preprint), Pathoumthong *et al.* (2023)
Orange	Bheemanahalli *et al.* (2021)
Soybean	Sakoda *et al.* (2019), Casado-García *et al.* (2020), Sultana *et al.* (2021)
Sundarbans (F)	Dey *et al.* (2023), Pathoumthong *et al.* (2023)
Tomato	Pathoumthong *et al.* (2023)
Turmeric	Andayani *et al.* (2020)
Wheat	Gibbs *et al.* (2021), Sun *et al.* (2021, 2023), Toda *et al.* (2021), Yang *et al.* (2021), Zhu *et al.* (2021), Pathoumthong *et al.* (2023)

**Table 3.** Overview of methods used to generate image data for deep learning analysis of stomata

Data collection type	Method	Paper
Non-destructive	Nail varnish-based surface impressions	Jayakody *et al.* (2017, 2021), Meeus *et al.* (2020), Millstead *et al.* (2020), Bheemanahalli *et al.* (2021), Costa *et al.* (2021), Cowling *et al.* (2021, Preprint), Gibbs *et al.* (2021), Hunt *et al.* (2021), Kwong *et al.* (2021), Razzaq *et al.* (2021), Ren *et al.* (2021), Toda *et al.* (2021), F. Zhang *et al.* (2022, 2023), Dey *et al.* (2023), Meng *et al.* (2023), Pathoumthong *et al.* (2023), Yang *et al*. (2024), Wang *et al*. (2024a)
	Direct microscope imagery	Bhugra *et al.* (2018), Li *et al.* (2019), Andayani *et al.* (2020), Song *et al.* (2020), Sun *et al.* (2021, 2023), Yang *et al.* (2021, 2024), Dai *et al.* (2022, Preprint), Liang *et al.* (2022), Sai *et al.* (2022, Preprint), Pathoumthong *et al.* (2023), Takagi *et al.* (2023)
Destructive	Epidermal separation	Casado-García *et al.* (2020), Aono *et al.* (2021), Zhu *et al.* (2021), Li *et al.* (2022), X. Zhang *et al.* (2023, Preprint), Yang *et al.* (2024)
	Use of leaf discs	Toda *et al.* (2018, Preprint)
	Freezing samples in liquid nitrogen	Bhugra *et al.* (2019)
	Leaf clearing	Fetter *et al.* (2019), Sultana *et al.* (2021)
	Optical topometry	Xie *et al.* (2021)
	Printing	Sakoda *et al.* (2019)

Whilst many of the studies focused on the task of counting stomata and estimating density, fewer extracted morphological traits, and even fewer performed comprehensive measurements of these traits (Table 4). Equally, despite a vast number of high-quality approaches to the detection and analysis of stomata, researchers have primarily focused on plant or species-specific implementations, with relatively few studies (e.g. Andayani *et al.,* 2020; Gibbs *et al.* 2021; Dey *et al.* 2023; Pathoumthong *et al*., 2023) combining datasets from multiple species.

**Table 4.** Overview of stomatal traits that have been estimated using deep learning methods and location of associated network code and datasets (where given)

	Image	Stomata					Stoma			Guard cell			Pore				Code/data availability
Publication	Class	Density	Index	Count	*g*_s_	*g*_smax_	Width	Length	Area	Width	Length	Area	Length	Class	Width	Area	
Jayakody *et al.* (2017)				x									x		x	x	On request
Toda *et al.* (2018, Preprint)													x	x	x	x	On request
Bhugra *et al.* (2018)		x		x									x	x	x		-
Fetter *et al.* (2019)				x													Tool available at: https://stomata.uvm.edu/
Sakoda *et al.* (2019)		x		x													On request
Li *et al.* (2019)				x									x		x	x	On request
Andayani *et al.* (2020)	x																-
Meeus *et al.* (2020)				x													Network code: Github Image data: Zenodo
Casado-García *et al.* (2020)		x		x			x	x									Model and datasets: Github
Millstead *et al.* (2020)																x	On request
Song *et al.* (2020)		x		x									x	x	x	x	-
Sultana *et al.* (2021)		x		x													On request Tool at: http://stomata.plantprofile.net
Kwong *et al.* (2021)		x		x													On request
Bheemanahalli *et al.* (2021)		x							x								-
Gibbs *et al.* (2021)	x	x		x		x				x	x	x	x		x	x	Model and datasets: Github
Aono *et al.* (2021)				x													Code and dataset: Zenodo
Ren *et al.* (2021)				x													-
Razzaq *et al.* (2021)				x										x			On request
Zhu *et al.* (2021)			x	x													-
Jayakody *et al.* (2021)				x					x								Network code: Github Data on request
Sun *et al.* (2021)		x		x	x											x	Network code: Github
Costa *et al.* (2021)		x		x										x		x	-
Yang *et al.* (2021)				x			x	x									Model and dataset: Github
Cowling *et al.* (2021, Preprint)		x		x													On request
Toda *et al.* (2021)		x		x			x	x									On request
Hunt *et al.* (2021)		x															-
Xie *et al.* (2021)		x		x			x	x	x								Dataset: Illinois data bank
Liang *et al.* (2022)													x		x	x	Trained model available at: http://plantphenomics.hzau.edu.cn/download_checkiflogin_en.action. Source code on request
Dai *et al.* (2022, Preprint)				x										x			-
Sai *et al.* (2023)													x		x	x	Network code: GitFront
Sun *et al.* (2023)							x	x	x								On request
F. Zhang *et al.* (2023)					x		x	x									Network code: Github
X. Zhang *et al.* (2023, Preprint)				x			x	x	x								-
Dey *et al.* (2023)	x						x	x									On request
Meng *et al.* (2023)		x		x												x	On request
Yang *et al*. (2024)		x		x			x	x								x	On request
Takagi *et al.* (2023)				x												x	Network code and datasets: Github and Zenodo
Li *et al.* (2023)		x		x										x			Data available on Zenodo
Pathoumthong *et al.* (2023)		x														x	Datasets available on Github
Wang *et al.* (2024a)		x		x			x	x	x	x	x	x					On request

### Object detection is sufficient for counts and classification but provides limited information on stomatal morphometry

You only look once (YOLO) networks are commonly chosen for object detection (i.e. the combination of localization and classification) due to their efficiency and accuracy. Indeed, for stomata detection, YOLO is the most common architecture used (Table 1).

YOLO is available in several versions, spanning the original network to the most recent YOLO-X. Most of these have been applied, or adapted, to stomata (e.g. Casado-García *et al.*, 2020; Ren *et al.*, 2021; Sultana *et al.*, 2021; Yang *et al.*, 2021; Dai *et al.*, 2022, Preprint; Zhang *et al.*, 2022; Li *et al.*, 2023). Example network adaptations include changes to the loss function (Ren *et al.*, 2021); adjustments to the network backbone to increase specificity (Zhang *et al.*, 2022); label smoothing to reduce overfitting and integration; and an attention mechanism, a layer to direct attention to specific parts of the data, to aid classification (Li *et al.*, 2023). Evaluation metrics differ between studies, but the majority report average precision or accuracy values exceeding 93%.

YOLO networks have been applied to a variety of different species including wheat (*Triticum aestivum*; Yang *et al.*, 2021; Zhang *et al.*, 2022), maize (*Zea mays* L.; Ren *et al.*, 2021; Yang *et al.*, 2021CJML_BIB_J_0048), barley (*Hordeum vulgare*; Casado-García *et al.*, 2020), beans (Casado-García *et al.*, 2020; Sultana *et al.*, 2021; Li *et al.*, 2023), and black poplar (Dai *et al.*, 2022, Preprint). A comparison of three versions of YOLO (v3, v4 and v5) applied to soybean (*Glycine max*) found that YOLOv5 was the most accurate but that YOLOv3 was the most time efficient, reflecting the common trade-off between time and accuracy for DL methods (Sultana *et al.*, 2021).

Whilst the majority of studies are specific to a single target plant species, LabelStoma (Casado-García *et al.*, 2020) aims to provide a more generalized model, enabling augmentations and transfer learning for new datasets, thus reducing the number of new images required. Furthermore, their published tool aims to make DL methods more accessible for less technical users via a user-friendly interface.

An alternative to the YOLO networks are region-based convolutional neural network (R-CNN) architectures, which, instead, use a two-stage approach. Single stage detection offers more efficient processing making it more suitable for real-time detection, but for the case in stomata, real-time processing speeds are generally not required. Cowling *et al.* (2021, Preprint) applied a Faster R-CNN to African rice (*Oryza glaberrima*), achieving a comparable accuracy scores to the YOLO-based methods. Similarly, a visual geometry group (VGG) is a standard deep CNN that specializes in localization and classification of objects, yielding comparable accuracy when applied to stomatal analysis (Sakoda *et al.*, 2019; Meeus *et al.*, 2020; Aono *et al.*, 2021).

With advances in hardware and in DL development, lightweight architectures, i.e. those capable of running on devices with less computational power such as handheld devices, have been generated. Kwong *et al.* (2021) use MobileNetv1 to estimate stomatal density in oil palm (*Elaeis guineensis*) and utilize image splitting to reduce the memory requirements of the network. Alternatively, Razzaq *et al.* (2021) combined MobileNetv2 with a single shot detector (SSD) for detection and classification of stomata within pre-processed images of quinoa (*Chenopodium quinoa*). This latter network has also been applied within a portable set-up consisting of a microscope feed directly connected to a Jetson Nano (a portable GPU; NVIDIA, Santa Clara, CA, USA), for real-time detection in wheat (Toda *et al.*, 2021). Together, these published methods present potential for an increase in the affordability and accessibility of DL methods, as well as more flexible and portable set-ups, which are likely to permit *in situ* analysis.

Applications of object detection-based methods are varied but often include counts and/or density; classification as open or closed; prediction of stomatal area via post-network image processing; width and height measurements; and estimates of stomatal conductance (Fig. 3; Table 4). However, object detection methods present limitations in regards to accuracy of obtaining morphological traits. For example, if stomata are not orientated along the horizontal or vertical axes, trait measurements may be distorted (Fig. 3C). To overcome this, an approach called RotatedStomataNet was proposed, which allows bounding boxes to have any rotation, ensuring a tighter fit around the stomata (Yang *et al*., 2024). Alternatively, image analysis methods have been applied; for example, histogram of gradients utilized by Toda *et al.* (2018, Preprint) in their method DeepStomata.

### Semantic methods provide more information of stomatal morphology

Semantic segmentation results in pixel-level classification of images. This permits the preservation of boundaries, or shapes, which, in turn, can lead to more in-depth trait analysis (Fig. 3B). Unlike object detection-based methods, these have often been used to segment pore and/or guard cells, thus permitting more precise area measurements. For example, over 30 stomatal traits including guard cell and stomatal area, length, width, and orientation, and stomatal evenness, divergence, and aggregation index can be yielded in the tool StoManager1, presented by Wang *et al.* (2024a). StoManager1 is based on a YOLO network that has been subsequently adapted to perform semantic segmentation.

Another popular semantic network is Mask-RCNN, which has been applied to numerous problems in stomata literature (Table 1; Song *et al.*, 2020; Bheemanahalli *et al.*, 2021; Costa *et al.*, 2021; Jayakody *et al.*, 2021; Sai *et al.*, 2022, Preprint; Meng *et al.*, 2023). Target species are varied including sorghum (*Sorghum bicolor*; Bheemanahalli *et al.*, 2021), sweet orange (*Citrus sineensis*; Costa *et al.*, 2021), black poplar (*Populus nigra*; Song *et al.*, 2020), Arabidopsis, and barley (Sai *et al.*, 2022, Preprint).

Similarly to many of the proposed object detection-based networks, adaptations have been applied to semantic networks to improve specificity for stomatal detection. For example, Jayakody *et al.* (2021) expanded on their previous work (Jayakody *et al.*, 2017), combining 16 datasets from 12 sources, to produce a more generic method for stomatal assessment using a Mask R-CNN. They proposed a three-stage approach to detecting stomatal boundaries, encompassing (i) pre-processing of images to remove colour space biases, which occur when images are captured in different conditions; (ii) estimation of the stomatal boundaries using a Mask R-CNN with transfer learning; and (iii) reduction in the number of false positives using a statistical filter based on the average stomatal size and confidence scores. The proposed method achieved an accuracy of 95.1%. Similarly, X. Zhang *et al.* (2023, Preprint) adjusted the U-Net architecture by altering the encoder, to introduce an attention mechanism, and fine-tuning the optimizer to detect stomata in lettuce (*Lactuca sativa*).

Whilst the majority of the reported papers present methods to extract traits, few have applied this to answering biological questions, such as determining the impact of irrigation of crop performance or predicting potential gas exchange capacity. Bhugra *et al.* (2019) used a combination of networks to investigate the impact of irrigation on rice cultivars by estimating count and density of stomata along with pore length, width, and area. Liang *et al.* (2022) investigated the opening and closure of maize stomata under varying levels of drought using time lapse imaging. Gibbs *et al.* (2021) proposed a method to automatically estimate stomatal morphometry (encompassing both guard cell and pore morphometry) in order to estimate anatomical maximum stomatal conductance (*g*_smax_, e.g. Franks and Beerling, 2009).

Modern microscopes permit the real time detection or analysis of stomata and so can be used to analyse patterns of opening and closing. This was proposed by Sun *et al.* (2021), alongside an easy-to-use interface, to study changes in stomatal aperture. Sun *et al.* (2023) subsequently improved this method and proposed *StomataTracker*, a tool to analyse the circadian rhythm (temporal pattern of opening and closing of stomata) applied to wheat. They captured videos of the wheat epidermis, which were then separated into their constituent frames for analysis. *StomataTracker* consists of a three-stage process: (i) multi object tracking using improved version of the simple online and real-time tracking (SORT) algorithm, which applies a lightweight detector (YOLOv3) to detect stoma and assign unique IDs; (ii) binary classification of each stoma as open or closed, which permits estimates of rest time and circadian rhythm; and (iii) semantic segmentation to obtain a mask image, enabling morphological traits, namely stomatal length, width, area, and perimeter, to be estimated (Sun *et al.*, 2023).

Less common are methods to estimate the stomatal index as they require the detection of both stomata and surrounding epidermal cells. This was addressed by Zhu *et al.* (2021), who obtained stomatal impressions of two wheat varieties. They utilized a Faster R-CNN model to count stomata, and a U-Net model to segment the epidermal cell network. Following post-processing steps to address artifacts in the cell network, they were able to estimate the number of epidermal cells and thus calculate stomatal index (Zhu *et al.*, 2021).

Whilst this review primarily focuses on stomatal morphometry analysis, additional literature on pavement cell segmentation is also worth noting. LeafNet (Li *et al.*, 2022) is one such example, where a DCNN was proposed for the detection of stomata and a region merging algorithm to segment the pavement cells in Arabidopsis. Comparisons with other pavement cell segmentation methods are also discussed (Li *et al.*, 2022).

### Alternative deep learning networks can overcome issues in datasets or provide an alternative route to phenotyping

Some published DL methods fail to classify as object detection or semantic, but still allow stomatal traits to be analysed. These have been applied to a variety of tasks including counting (Fetter *et al.*, 2019; Hunt *et al.*, 2021), species identification (Andayani *et al.*, 2020; Dey *et al.*, 2023), and data improvement (Bhugra *et al.*, 2018). For example, Fetter *et al.* (2019) developed *StomataCounter*, a DCNN based on AlexNet, to estimate stomatal count; it was trained using four datasets. As opposed to a bounding box detection, the DCNN produced a heatmap of potential stomata, with 94% accuracy when applied to unseen species, indicating generalization of the method.

Bhugra *et al.* (2018) proposed a 13-layer CNN for the detection and segmentation of pores in rice. They focused on the recovery of missing information caused by occlusions by using an inpainting algorithm to fill in the missing data. Their proposed method addresses many of the challenges experienced when working with microscopic images of surface impressions. Features such as image artefacts, overlapping epidermal structures such as trichomes or papillae, feature-rich backgrounds, and small stomatal sizes make analysis challenging. Other challenges include presence of dust or air bubbles, and blur within images, which can similarly be addressed using DL approaches (Jayakody *et al.*, 2021).

## Current limitations of deep learning methods and future directions

Literature often reports that the bottleneck in plant analysis and improvement arises due to long timeframes associated with phenotyping. Recent interest in DL methods, such as those presented here, has led to a great reduction in these timeframes. However, a bottleneck now exists in relation to the availability of datasets, and the ability to equally evaluate methods. DL models require an initial annotated dataset for training, which can be time-consuming, expensive, and the generation of image data can lead to large storage requirements. In addition, variability in the dataset will determine how generalized it is, and thus what other datasets it can be applied to; i.e. a dataset encompassing only a single species captured using a single set up is unlikely to be applicable to another species or set-up, unless similar; a DL model can only ‘see’ what it has ‘seen’ before.

Variations exist in the pipeline used to generate and analyse data on stomata, encompassing all steps from data acquisition to post-processing. For example, for the data acquisition stage, Pathoumthong *et al.* (2023) indicate improved efficiency of using a handheld microscope over nail varnish based surface impressions. However, they did not identify a trend as to which acquisition method produced better overall estimates of morphology, suggesting species and case specific benefits to each method. Therefore, further work is required to determine the optimal pipeline for each species and physiological aim.

Despite the capabilities of DL methodologies, they are not applicable to a wide variety of situations and, as such, there still remains a bottleneck in their development. In part this could be addressed through the use of GANs, which can be used to generate artificial datasets and thus increase the amount of available data (Goodfellow *et al.*, 2014, Preprint; Creswell *et al.*, 2018). Future methods also require development of techniques to accurately and appropriately evaluate the proposed networks. For example, Dey *et al.* (2023) performed an empirical comparison of nine deep learning models for the identification of stomata from 11 different tree species, spanning eight families. They introduced a normalized leverage factor, which combines accuracy, precision, recall, and F1-score to create a more uniform evaluation function to rank approaches. However, in order to advance and facilitate widespread and rapid stomatal analysis, more shared resources need be made available. Pipelines require alternative steps to ensure that they are more generic.

Future research directions require advancements in terms of the biological implications of the results, with a move away from object detection based methods towards semantic segmentation, instance segmentation, and real-time detection and monitoring of stomatal behaviours. There is also a need for the exploration of the 3-dimensional (3D) traits of the stomatal structure, using data collected from sources such as confocal microscopy, optical tomography, and surface topography measurements (Thompson *et al.*, 2017; Xie *et al.*, 2021; Davaasuren *et al.*, 2022). Initial attempts have been made towards this goal. Optical tomography was applied by Xie *et al.* (2021) to acquire a 3D model of the leaf epidermis of maize. Their pipeline involved multiple steps, initially flattening the 3D model into a single 2D image using Gaussian filters and then employing a mask R-CNN architecture to segment the stomata and pavement cells. From this, stomatal density, width, length, and area were estimated, but 3D information was lost.

Despite the extensive research on stomatal biology, current knowledge is poorly translated into the context of field experimentation. This stage will be integral for future yield improvement strategies. This is partly due to the nature of the publications; the majority are targeted as method development, with very few applying the proposed method towards answering a biological question.

However, a number of caveats arise from advancing DL methods for application in biological analysis. Whilst DL architectures become more advanced in terms of their capabilities, there is still a need to ground-truth these generated measurements with actual conductance measurements in order to accurately correlate the results with physiological function. This step cannot be underestimated, potentially requiring modification of gas exchange equipment for simultaneous capture of stomatal apertures. Care must also be taken over which method is used to capture the required data on stomatal complexes, for example restricting the use of leaf impressions for density measurements as they do not permit sufficient resolution for analysis of dimensions (Matthaeus *et al.*, 2020).

Thus, despite the potential capabilities of semantic based methods, it may be that biologically relevant or useful information is currently limited towards more basic phenotypic extraction such as density, which can be readily obtained using object detection-based methods. For example, in one of the few published works that link stomatal structure to function, Hunt *et al.* (2021) investigated the impact of manipulating light and CO_2_ concentration on stomatal density and conductance of barley. Whilst they measured stomatal conductance using a gas exchange system, density was estimated through DL via custom CNNs to locate and then classify image crops as to whether they contain stomata.

Moving forward, it is encouraged to advocate a stronger link between computer scientists and biologists and expand beyond stomatal detection to instead produce methods that reliably measure multiple traits. A global dataset will help to eliminate this redundancy and improve effectiveness and efficiency. As such, we have generated StomataHub (www.stomatahub.com), a free online resource to encourage collaborations and the sharing of datasets. We hope that StomataHub, or other similar resources such as that produced by Wang *et al.* (2024b) (encompassing a dataset of 11 000 annotated hardwood images), will address this and provide a free open-source approach moving forwards.

## Conclusion

In conclusion, DL provides a promising approach for plant phenotyping tasks. Here we have presented details of the 43 published works to date that apply DL to the analysis of stomata. We discussed the variation in the pipeline required, from data generation through to post-processing analysis, and described some of the major networks that have been applied. Whilst the species studied and measurements generated are diverse, current restrictions lie in the availability of data, evaluation of methods, and generalization of different studies. Future advances will therefore require a shared global effort in providing datasets, innovations to link the corresponding phenotypic measurements to underlying physiology, as well as enhanced collaboration between biologists and computer scientists.

## Data Availability

No new data were generated in the production of this review.

## Abbreviations:

CNN: convolutional neural network
DL: deep learning
FN: false negative
FP: false positive
GAN: generative adversarial network
R-CNN: region-based convolutional neural network
TN: true negative
TP: true positive
VGG: visual geometry group
YOLO: you only look once.
